# The relationship between ischaemic brain lesions and cognitive outcome after aneurysmal subarachnoid haemorrhage

**DOI:** 10.1007/s00415-019-09408-8

**Published:** 2019-06-03

**Authors:** I. M. C. Huenges Wajer, M. E. Hendriks, T. D. Witkamp, J. Hendrikse, G. J. E. Rinkel, J. M. A. Visser-Meily, M. J. E. van Zandvoort, M. D. I. Vergouwen, J. B. de Vis

**Affiliations:** 1 Department of Neurology and Neurosurgery, G03.232, Brain Center Rudolf Magnus, University Medical Center Utrecht, Utrecht University, Heidelberglaan 100, 3584 CX Utrecht, The Netherlands; 2 Department of Radiology, University Medical Center Utrecht, Utrecht University, Utrecht, the Netherlands; 3 Department of Rehabilitation, University Medical Center Utrecht and Center of Excellence in Rehabilitation Medicine, Utrecht University, Rehabilitation Center de Hoogstraat, Utrecht, The Netherlands; 4 Experimental Psychology, Helmholtz Institute, Utrecht University, Utrecht, The Netherlands; 5 National Institute of Health, National Institute of Neurological Disorders and Stroke, Bethesda, MD USA

**Keywords:** Subarachnoid haemorrhage, Aneurysm, Cerebral ischaemia, MRI, Cognition

## Abstract

**Background:**

Cerebral ischaemia is thought to be an important determinant of cognitive outcome after aneurysmal subarachnoid haemorrhage (aSAH), but the exact relationship is unclear. We studied the effect of ischaemic brain lesions during clinical course on cognitive outcome 2 months after aSAH.

**Methods:**

We studied 74 consecutive patients admitted to the University Medical Center Utrecht who had MRI post-coiling (3–21 days post-aSAH) and neuropsychological examination at 2 months. An ischaemic lesion was defined as hyperintensity on T2-FLAIR and DWI images. We measured both cognitive complaints (subjective) and cognitive functioning (objective). The relationship between ischaemic brain lesions and cognitive outcome was analysed by logistic regression analyses.

**Results:**

In 40 of 74 patients (54%), 152 ischaemic lesions were found. The median number of lesions per patient was 2 (1–37) and the median total lesion volume was 0.2 (0–17.4) mL. No difference was found between the group with and the group without ischaemic lesions with respect to the frequency of cognitive complaints. In the group with ischaemic lesions, significantly more patients (55%) showed poor cognitive functioning compared to the group without ischaemic lesions (26%) (OR 3.4, 95% CI 1.3–9.1). We found no relationship between the number and volume of the ischaemic lesions and cognitive functioning.

**Conclusions:**

Ischaemic brain lesions detected on MRI during clinical course after aSAH is a marker for poor cognitive functioning 2 months after aSAH, irrespective of the number or volume of the ischaemic lesions. Network or connectivity studies are needed to better understand the relationship between location of the ischaemic brain lesions and cognitive functioning.

## Introduction

Patients who survive an episode of aneurysmal subarachnoid haemorrhage (aSAH) often show good physical recovery, but many have cognitive complaints or impairments that hamper complex activities in daily life, such as work and social participation [1–4].

Cerebral ischaemia plays a role in poor cognitive outcome after aSAH [5-7]. Besides hypoperfusion during aneurysmal rupture [8], cerebral ischaemia can also be a result of treatment of the aneurysm [9] or delayed cerebral ischaemia (DCI) [10]. Most previous studies on the relationship between cerebral ischaemia and cognition after aSAH tried to distinguish cerebral ischaemia due to the initial aSAH from delayed cerebral ischaemia (DCI) and, therefore, focused on cerebral ischaemia in the acute phase (< 72 or < 96 h)[11, 12] or in late (chronic) phase [3, 11, 13–15]. Moreover, these studies only included cognitive functioning and left out patient’s own (subjective) experience about their cognitive abilities. As a result, the exact relationship between cerebral ischaemia and cognitive outcome is not completely clear.

Insight in the relationship between ischaemic brain lesions (regardless of its underlying aetiology) and cognition might help predict patients’ cognitive outcome after aSAH and help clinicians to decide if a neuropsychological assessment and cognitive rehabilitation are indicated. The aim of the present study is to examine whether aSAH patients with ischaemic brain lesions have an increased risk of poor cognitive outcome.

## Methods

### aSAH patients

This study was approved by the Medical Ethics Review Committee of the University Medical Center of Utrecht (UMCU). Patients were retrieved from a prospectively collected series of aSAH patients admitted to the UMCU between November 2012 and February 2016 and, as part of a study protocol, had MRI between coiling and discharge. In the UMCU, all patients discharged home or to a rehabilitation institution are invited for our routine outpatient clinic around 2 or 3 months post-aSAH. At this outpatient clinic, a nurse practitioner specialized in SAH asked patients about their cognitive complaints (questionnaire) and a trained neuropsychologist examined a neuropsychological examination (NPE). Inclusion criteria were: (1) SAH, defined as the presence of subarachnoid blood as shown by CT or lumbar puncture, (2) aneurysmal cause of the SAH as determined by the visualization of an aneurysm on either CT angiography, magnetic resonance angiography (MRA), or digital subtraction angiography, (3) MRI performed between coiling and discharge and (4) patient’s visit to the outpatient clinic around 2 or 3 months post-aSAH.

### Demographics, premorbid functioning and aSAH characteristics

Demographic data included sex, age and level of education. The level of education was classified using a Dutch classification system ranging from 1 [did not complete primary school] to 7 [university degree] [16]. Pre-morbid level of functioning was enquired using the 10-item version of the Barthel-Index. Moreover, the presence of pre-SAH neurological diagnoses was investigated based on patients’ medical record. The aSAH characteristics were: (1) clinical condition on admission and (2) location of the aneurysm. The clinical condition on admission was graded using the Prognosis on Admission after Aneurysmal Subarachnoid Haemorrhage (PAASH) [17]. A PAASH rating of I–III (Glasgow Coma Scale score 8–15) was classified as good clinical condition and a PAASH rating of IV–V (Glasgow Coma Scale score 3–7) as poor clinical condition.

### Radiological characteristics

The amount of subarachnoid blood was rated on the diagnostic computed tomography (CT) scan (i.e. the first CT scan after ictus) by J.B.D.V. (3 years of experience with CT imaging) using the Hijdra score [18]. MRI was performed at either a 1.5 T or a 3 T scanner (Intera/Achieva, Philips Medical Systems, Best, The Netherlands) with a quadrature body coil for transmission and an eight-channel head coil as a signal receiver. The MR imaging protocol included a T1- and T_2_-weighted imaging sequence, a time-of-flight MR angiography, a T_2_-weighted fluid-attenuated inversion recovery (T_2_-FLAIR) sequence and a diffusion-weighted imaging (DWI) sequence. The scan parameters of the T_2_-FLAIR sequence were TR/TI/TE = 10,000/2800/140 and 10,000/2800/120 ms, voxel size 0.7 × 0.7 × 5 mm^3^ and 0.4 × 0.4 × 4  mm^3^, and slice gap 1 mm. The scan parameters of the DWI sequence were TR/TE = 3348/98 and 3015/68 ms, b-factors = 2 (0 and 1000 s/mm^2^), voxel size = 1 × 1 × 5 mm^3^ and 0.5 × 0.5 × 4 mm^3^, and a slice gap of 1 mm. Ischaemic brain lesions were defined as hyperintensity on the T_2_-FLAIR and DWI. The T_2_-FLAIR and DWI image quality, number of ischaemic brain lesions and lesion location were evaluated simultaneously by a neuroradiologist (T.D.W., 30 years of experience with neuroradiology), who was blinded to the patients’ information. The volume of the ischaemic brain lesions was measured through manual segmentation on either the FLAIR or DWI images by M.E.H., also blinded to the patients’ information, using Picture Archiving and Communication System (PACS) software (Sectra AB, Linköping, SWEDEN).

### Cognitive outcome

Cognitive outcome was measured both as cognitive complaints (subjective) and as cognitive functioning (objective) 2 months post-SAH. Cognitive complaints were identified using the 13 cognitive items of the Checklist for Cognitive and Emotional Consequences following stroke (CLCE-24) [19]. Based on patient’s answers on the interview at the SAH outpatient clinic, the interviewer scored a ‘0’ for absence and a ‘1’ per item for the presence of complaints; the sum score indicates the number of experienced complaints.

Cognitive functioning was measured using a neuropsychological examination consisting of 13 (sub)tests covering six main cognitive domains: language, memory, attention, executive functioning, processing speed and visuospatial functioning. A shortened (15-item) version of the Boston Naming Test (BNT) was used to evaluate language. Memory was assessed by the Digit Span of the Wechsler Adult Intelligence Scale III (WAIS-III), the immediate recall and delayed recall scores of the Rey Auditory Verbal Learning Task-Dutch version (RAVLT-D), the delayed Rey–Osterrieth Complex Figure Test (Rey-CFT) and Category Fluency (using animals). A shortened version of the Visual Elevator subtest of the Test of Everyday Attention (TEA) was administered to measure attention. Executive functioning was measured using the Letter Fluency (‘A’), Key Search of the Behavioural Assessment of Dysexecutive Syndrome (BADS) and the go/no go subtest of the Frontal Assessment Battery (FAB). Processing speed was assessed using the Digit Symbol Coding of the WAIS-III. To evaluate visuospatial functioning, we used Judgement of Line Orientation Test (JLO) and the copy score of the Rey-CFT.

### Analyses

The number of cognitive complaints was dichotomized, based on the median split of the sum score of the CLCE-24 (3 cognitive complaints). Raw test scores from the individual neuropsychological tests were transformed into percentile scores based on norm scores of each test. If applicable corrections were made based on sex, age and/or level of education. Scores below the 5th percentile were considered as indicating an impaired cognitive performance. The differences in the amount of impairments on each neuropsychological test were analysed by Chi-square test. Next, the number of impaired tests per individual was determined and dichotomized based on the median split (> 2 impaired test scores defined as poor cognitive functioning). Descriptive analyses were used to describe baseline and imaging characteristics. The relationship between ischaemic brain lesions (presence, number, volume) and cognitive complaints as well as the relationship between ischaemic brain lesions (presence, number, volume) and cognitive functioning was investigated by logistic regression analysis. Because the clinical condition on admission after aSAH is related to cerebral circulation in the acute phase after aSAH [8], no adjustment for clinical condition on admission was made. Analyses were checked for collinearity. We considered a *p* value < 0.05 as statistically significant.

## Results

### aSAH population

Ninety-four patients were eligible for inclusion in this study. Of these 94 patients, 20 patients did not complete the NPE because of pain (1), fatigue (1), emotionality during the assessment (2), insufficient proficiency of Dutch language (3), aphasia (2), lack of motivation (2) or lack of time (5). The remaining 74 patients were included in the analyses. Characteristics of the included patients are listed in Table 1. All patients were pre-morbid functionally independent (Barthel Index ≥ 18.) In total, 3 patients were diagnosed with minor stroke/TIA before the aSAH and none of the patients was diagnosed with pre-SAH MCI or dementia.

**Table 1:** Characteristics of aSAH patients (*N* = 74)

*Demographic characteristics*	
Sex	
Female (*n*, %)	57 (77%)
Age at time of bleeding (years; mean, SD)	56 ( ± 10)
Level of education (n, %)	
Low (Verhage 1–3)	12 (16%)
Middle (Verhage 4–5)	46 (62%)
High Verhage 6–7)	16 (22%)
*Clinical characteristics*	
Time between MRI and aSAH (days; mean, range)	12 (3–21)
Time between aSAH and NPE (days; mean, range)	60 (24–109)
Clinical condition on admission (PAASH) (*n*, %)	
Good	71 (96%)
I = GCS 15	44 (60%)
II = GCS 11–14	26 (35%)
III = GCS 8–10	1 (1%)
Poor	3 (4%)
IV = GCS 4–7	1 (1%)
V = GCS 3	2 (3%)
*Imaging characteristics*	
Amount of subarachnoid blood (Hijdra score) (Median, range)	
Blood in basal cisterns	24 (0–30)
Blood in ventricles	2 (0–9)
Total	26 (0–39)
Aneurysm location (*n*, %)	
Anterior circulation	64 (86%)
Acom/A1/pericallosal artery	41 (55%)
MCA	0 (0%)
ICA/Pcom	23 (31%)
Posterior circulation	10 (14%)
PCA	1 (1%)
Basilar artery	7 (9%)
Vertebral arteries/PICA/AICA	2 (3%)
Location cerebral ischaemia (n, %)	
Left	14 (19%)
Right	14 (19%)
Both	12 (16%)
Anatomical areas (number and % of all ischaemic lesions)	
Cortical	76 (50%)
Subcortical	61 (40%)
Internal capsule	5 (3%)
Basal ganglia	6 (4%)
Cerebellum	4 (3%)
Brainstem	0 (0%)
Perfusion territories (number and % of ischaemic lesions)	
ACA	26 (17%)
MCA	32 (21%)
PCA	10 (7%)
Basilar artery, vertebral artery and PICA	7 (5%)
Watershed areas	77 (51%)

*aSAH* aneurysmal subarachnoid haemorrhage, *MRI* magnetic resonance imaging, *NPE* neuropsychological examination, *PAASH* prognosis on admission of aneurysmal subarachnoid haemorrhage, *GCS* glasgow coma scale, *Acom* anterior communicating artery, *MCA* middle cerebral artery, *ICA* internal carotid artery, *Pcom* posterior communicating artery, *PCA* posterior cerebral artery, *PICA* posterior inferior cerebellar artery, *AICA* anterior inferior cerebellar artery *ACA* anterior cerebral artery

### Ischaemic brain lesions

In 40 of 74 patients (54%), 152 ischaemic lesions were found, of which 4 were old lesions (before aSAH). In the subgroup of 40 patients with ischaemic lesions, the mean number of lesions per patient was 4 (median 2, range 1–37) and the mean total lesion volume was 1.6 mL (median 0.2, range 0–17.4). The location of the ischaemic brain lesions is shown in Table 1.

### Cognitive outcome

Two months post-SAH, sixty-one patients (82%) reported at least one cognitive complaint. The frequencies of the different cognitive complaints are shown in Table 2.

**Table 2:** Frequencies of cognitive complaints (CLCE-24)

	*N*	%
Keeping up; has become slower	46	62
Attending to things	42	57
Remembering new information	40	54
Doing two things at once	31	42
Planning and organizing things	23	31
Taking initiative	20	28
Performing daily activities	20	28
Remembering old information	6	8
Perceiving time	5	7
Orientating to places or persons	3	4
Speaking or writing	1	1
Social aspects of language	1	1
Attending to a part of the body or space	1	1

Table 3 shows the results of the separate cognitive tests. Taking all neuropsychological tests scores together, nine patients (12%) showed no impaired test scores. Thirty-four patients (46%) had impairments (≤ 5th percentile) on one or two of the subtests. Forty-nine patients (42%) were impaired on more than two subtests of the NPE.

**Table 3:** Frequencies of impaired test scores on neuropsychological examination 2 months post-SAH (total *n* = 74) including Chi-square analyses to differences between patients with ischaemic lesions (*n* = 40) and patients without ischaemic lesions (*n* = 34)

Cognitive domain	Test	Impaired test score (< 5th percentile)						
		Ischaemic lesions		No ischaemic lesions		*p*	Total	
		*N*	%	*N*	%		*N*	%
Language	BNT	12	30	4	12	.06	16	22
Memory	Digit span	6	15	5	15	.94	11	15
	RAVLT direct	17	43	9	27	.15	26	35
	RAVLT delayed	18	45	10	29	.17	28	38
	Rey-CFT delayed	8	20	3	9	.16	11	15
	Category Fluency	3	8	0	0	.10	3	4
Attention and executive functioning	Visual elevator score	6	15	4	12	.67	10	14
	Visual elevator time	9	23	6	18	.59	15	20
	Letter fluency	8	20	4	12	.30	12	16
	Key search	6	15	5	15	.78	11	15
	Go-no go	6	15	8	24	.44	14	19
Processing speed	Digit symbol coding	5	13	3	9	.53	8	11
Visuospatial	JLO	4	10	5	15	.84	9	12
functioning	Rey-CFT copy	9	23	7	21	.80	16	22

*BNT* Boston naming test, *RAVLT* Rey Auditory Verbal Learning Task, *Rey-CFT* Rey–Osterrieth Complex Figure Test, *JLO* judgment of line orientation

### Relationship between ischaemic parameters and cognitive outcome

Neither the presence of ischaemic lesions (OR 1.46, 95% CI 0.58–3.70) nor the number (OR 1.12, 95% CI 0.92–1.16) or volume of the ischaemic lesions (OR 1.00, 95% CI 1.00–1.00) was significantly related to the frequency of cognitive complaints.

Regarding the NPE, the group of patients with ischaemic lesions showed more impaired tests scores on most of the neuropsychological tests, except in the go/no go subtest of the FAB and the JLO it was unexpectedly the other way around. However, none of the differences in frequencies of impaired tests scores were significant. After summarizing all neuropsychological tests, of the 40 patients with ischaemic lesions, 22 (55%) had poor cognitive functioning, whereas in the 34 patients without ischaemic lesions nine patients (26%) had poor cognitive functioning (OR 3.40, 95% CI 1.27–9.08). Within the subgroup of patients with ischaemic brain lesions, the number (OR 1.03, 95% CI 0.91–1.17) and volume (OR 1.00, 95% CI 1.00–1.00) of the ischaemic lesions were not related to cognitive functioning.

## Discussion

In aSAH patients with relatively good functional outcome, those with ischaemic brain lesions during clinical course have an increased risk of poor cognitive functioning 2 months post-SAH, compared to those without ischaemic brain lesions. Although the presence of ischaemic brain lesions was clearly related to an increased risk of developing cognitive deficits, within the group of patients with ischaemic brain lesions there was no relation between the number and volume of ischaemic lesions and cognitive functioning.

The absence of a relationship between the number and volume of ischaemic lesions and cognitive functioning is in line with the findings of the only previous study we found with the relationship between ischaemic lesions and cognitive functioning using number and volume of ischaemic lesions in their analyses [12]. In a study that examined infarct load after aSAH by the number of regions in which cerebral ischaemia was located, the number of regions involved was related to cognitive functioning [5]. Although that study only focused on DCI, this suggests that not the number or volume of the ischaemic lesions, but the distribution of the ischaemic lesions, is an important factor of the variability in cognitive functioning after aSAH.

The lack of a relationship between the number and volume of the ischaemic lesions and cognitive functioning after aSAH in our study might also be a result of the relative low ischaemic load found in our sample, which is possibly a consequence of the selection of patients in our study. Although MRI is more sensitive in detecting cerebral ischaemia after aSAH than CT [20], the use of MRI has led to a selection of relatively good patients, since MRI is not always feasible in patients with a poor clinical condition. Moreover, we only included patients who were discharged home or to rehabilitation center with intention to be transferred home in 2 months. Although patients in our sample had a relatively good functional outcome, they did experience considerable cognitive complaints and cognitive impairments. Despite the relatively low ischaemic load, we found an effect of ischaemic brain lesions on cognitive functioning, indicating a robust relationship.

Because of limited power in our sample, we did not perform a subgroup analysis on the location of ischaemic lesions. The location of the ischaemic lesions might, however, be important for the nature and severity of the cognitive impairments. Small brain lesions can disturb hubs and reduce the integrity of functional brain networks which is related to cognitive decline [21]. Moreover, our study cannot give any judgement about the aetiology of the ischaemic lesions that are found, because of variance in the timing of the post-SAH MRI and the absence of a pre-aSAH MRI.

In contrast to cognitive functioning, we found no relation between ischaemic brain lesions and cognitive complaints after aSAH. This discrepancy confirms that this subjective measure of cognition reflects another construct compared to objective measures of cognitive outcome. Only a small proportion of the cognitive complaints can be explained by potentially underlying cognitive impairments [1].

Based on the present study, clinicians should be extra attentive to cognitive impairments when, during the clinical course after aSAH, one or more ischaemic lesions are present on MRI. Network or connectivity studies are needed to better understand the relationship between location of the ischaemic brain lesions and cognitive functioning. Future studies on the effectiveness of interventions to prevent cerebral ischemia after aSAH should not only focus on functional outcome but also take effects on cognitive outcome into account.
